# The role of the cell wall compartment in mutualistic symbioses of plants

**DOI:** 10.3389/fpls.2014.00238

**Published:** 2014-06-02

**Authors:** Mélanie K. Rich, Martine Schorderet, Didier Reinhardt

**Affiliations:** Department of Biology, University of FribourgFribourg, Switzerland

**Keywords:** symbiosis, *Petunia*, *Rhizophagus*, *Rhizobium*, arbuscular mycorrhiza, nodulation, cell wall, symbiotic interface

## Abstract

Plants engage in mutualistic interactions with microbes that improve their mineral nutrient supply. The most wide-spread symbiotic association is arbuscular mycorrhiza (AM), in which fungi of the order *Glomeromycota* invade roots and colonize the cellular lumen of cortical cells. The establishment of this interaction requires a dedicated molecular-genetic program and a cellular machinery of the plant host. This program is partially shared with the root nodule symbiosis (RNS), which involves prokaryotic partners collectively referred to as rhizobia. Both, AM and RNS are endosymbioses that involve intracellular accommodation of the microbial partner in the cells of the plant host. Since plant cells are surrounded by sturdy cell walls, root penetration and cell invasion requires mechanisms to overcome this barrier while maintaining the cytoplasm of the two partners separate during development of the symbiotic association. Here, we discuss the diverse functions of the cell wall compartment in establishment and functioning of plant symbioses with the emphasis on AM and RNS, and we describe the stages of the AM association between the model organisms *Petunia hybrida* and *Rhizophagus irregularis*.

## INTRODUCTION

The plant cell wall is the outermost border of the plant body, and therefore the interface for interactions with the biotic and abiotic environment. Water and nutrients taken up from the soil have to cross the cell wall of the epidermis and the tissues between the epidermis and the vascular stele before they can enter the xylem for transport to the shoot with the transpiration stream (Kim et al., 2014). Together with the internal turgor pressure, the cell walls confer to the plant organs their particular shape (Zonia and Munnik, 2007), and in the case of dead tissues (xylem, wood), the walls represent the only remnant of the cells.

In interactions with pathogenic microbes, the cell wall is the first line of defense which provides a high level of general (non-host) resistance to plants. However, in mutualistic interactions such as root nodule symbiosis (RNS) and arbuscular mycorrhiza (AM), the cell walls of the host have to give way to the microbial partner in order to establish the intimate interfaces for developmental coordination and nutrient exchange. Intracellular accommodation of microbial symbionts thus involves dedicated pathways that evolved since the occurrence of the first symbiotic land plants over 400 Ma ago (Parniske, 2000; Brundrett, 2002). While the molecular-genetic program involved in mutual recognition and communication between the partners has recently been elucidated in considerable detail based on intense genetic and genomic analysis (Gutjahr and Parniske, 2013; Oldroyd, 2013), the later stages involving cellular coordination and establishment of the symbiotic interface are less well understood.

Here, we review recent developments in the domain of plant symbioses with the emphasis on the role of the cell wall compartment during penetration and establishment of the symbiotic interface. We discuss classical concepts and recent insight from genetics, genomics, and transcriptomics analysis concerning the mechanisms involved in symbiotic signaling, cell wall loosening, and penetration, and in the nutrition of the microbial endosymbiont. Finally, we provide a detailed description of the infection process in AM, the oldest and most widespread symbiotic association of plants, with the example of the interaction between *Petunia hybrida* and *Rhizophagus irregularis*.

## ANATOMY OF PLANT ROOTS

The cell walls of roots have to comply with two very different tasks. On the one hand, the root is dedicated to the acquisition of water and mineral nutrients. For this, the epidermis (the epidermis of the root) has to be maximally permeable. On the other hand, the root surface has to be protected from harmful microbes (pathogens), toxic solutes, and, under conditions of drought, water loss. To comply with these two seemingly contradictory requirements, the root has specialized cell layers for nutrient absorption and protection.

**FIGURE 1:**
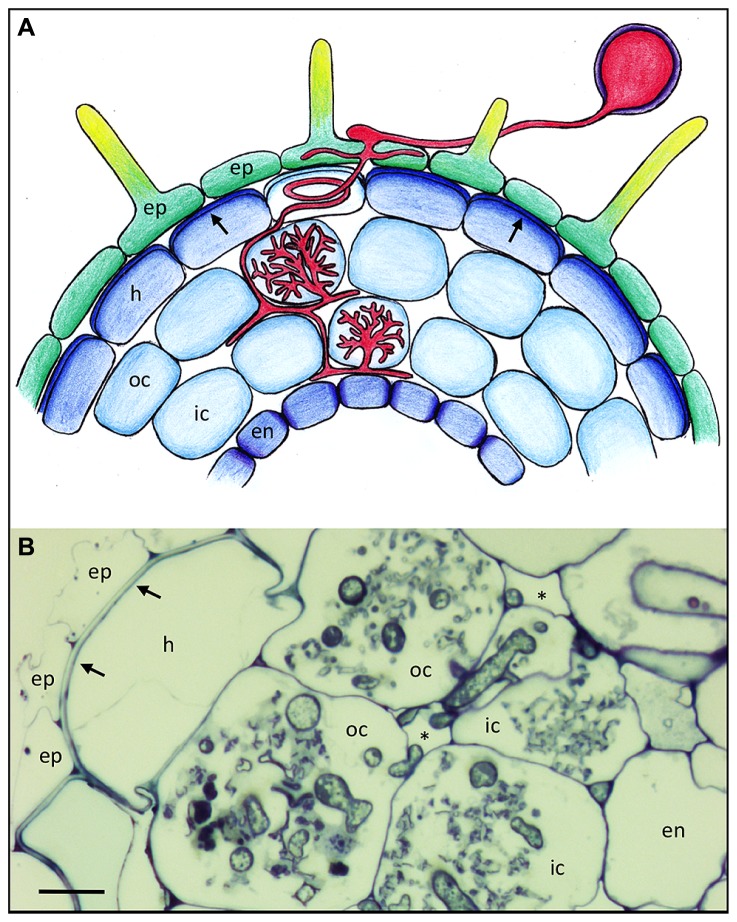
**Basic root anatomy and colonization pattern of the arbuscular mycorrhizal fungus *Rhizophagus irregularis* in *Petunia hybrida*. (A)** Schematic representation of a *Petunia* root with the different cell types and with an AM fungus (red). Arrows point to the reinforced outer cell wall of the hypodermis. Lighter coloration of the penetrated cell signifies a hypodermal passage cell. **(B)** Cross section of a mycorrhizal *Petunia* root. Arrows indicate the thick outer cell walls of the hypodermis. Asterisks indicate the intercellular air space between cortical cells. Fungal colonization is visible only in the cortex. ep, epidermis; h, hypodermis; oc, outer cortex; ic, inner cortex; en, endodermis. Size bar 10 μm.

With its thin cell walls, and the root hairs as surface extensions (**Figure 1A**), the epidermis is optimally suited for efficient nutrient uptake. In addition, the surface of the root system is increased by repeated lateral root formation and by the formation of tubular extensions of the epidermal cells, the root hairs. Further inside the root, the endodermis with its tight Casparian strip seals the vascular tissues in the stele from the cortex (Geldner, 2013b; **Figure 1A**). Water and solutes therefore have to pass through the endodermal cell layer exclusively symplastically, allowing this cell layer to control and filter the nutrient solution before it enters the xylem for the transfer to the shoot.

Many plant species have a second protective cell layer below the epidermis, referred to as hypodermis, that is often reinforced with thickened cell walls and hydrophobic impregnations (**Figure 1A**; Meyer and Peterson, 2013), and which can have a Casparian strip like the endodermis (Enstone et al., 2002; Schreiber, 2010), in which case it is referred to as exodermis (Meyer and Peterson, 2013). Hence, like the endodermis, the hypodermis can contribute to the filtering of the soil solution, and to protection of the root from pathogens. Some plants have a so-called dimorphic hypodermis that consists of two cell types, the predominant impregnated cell type and individual interspersed cells that are characterized by higher permeability than their neighbors (Shishkoff, 1987). These are thought to function as dedicated passage cells for water and solutes, and interestingly, these cells are the preferred entry points for AM fungi. (Sharda and Koide, 2008). After they have passed beyond the hypodermis, AM fungi colonize the cortex by colonizing individual cells with their arbuscules (**Figures 1A,B**).

Besides the evolutionary differences in root structures between plant taxa (Shishkoff, 1987), a single species can have a heterogeneous root system. For example, rice first forms large crown roots (CR) from which large lateral roots (LLR) emanate, which in turn carry small lateral roots (SLR; Gutjahr and Paszkowski, 2013). AM colonization in rice is restricted to CR and LLR, presumably because SLRs lack a cortex and therefore cannot host AM fungi (see The Infection Process: Overcoming the Cell Wall).

## STRUCTURE OF CELL WALLS IN ROOTS

In general, plant cell walls are assemblies of polysaccharides of different composition and properties. The main components are cellulose, hemicelluloses, and pectins. The cellulose component is chemically homogenous and consists of linear β-1,4-linked glucose moieties. Individual linear cellulose molecules are assembled to thicker cellulose microfibrils that represent the primary load-bearing component of the cell wall (McFarlane et al., 2014). The cellulose microfibrils are interlinked with hemicelluloses that consist of a linear polymer of glucose (like cellulose), but with heterogeneous side chains made of various sugars such as xylose, arabinose, and others (Scheller and Ulvskov, 2010). This complex three-dimensional network is embedded in a matrix of pectins, that represent highly acidic linear polymers of primarily galacturonic acid, which is interlinked by complexation with calcium (Caffall and Mohnen, 2009). While cellulose is formed in situ at the plasma membrane by the cellulose synthase complex (McFarlane et al., 2014), hemicelluloses and pectin are produced in the Golgi apparatus and transported to the apoplast through vesicular trafficking. Besides these carbohydrate polymers, cell walls contain variable amounts of structural proteins and secondary modifications such as impregnation with lignin and suberin for physical reinforcement and increased water impermeability. While the aerial parts of the plant (leaves and stems) have an external cuticle layer as a transpiration barrier and as a protection against biotic and abiotic agents, many roots have two internal barriers, the endodermis and the hypodermis (see above; Schreiber, 2010).

While the endodermis is a universal feature of the roots of land plants, the nature of the hypodermis is very variable among the higher plants. Whereas some xerophytes have highly reinforced hypodermal cells that serve as a secondary cuticle, the hypodermis of other species like for example *Arabidopsis thaliana* represents a normal cortex layer without specialized cell wall features. *Petunia* takes an intermediate position with a distinguished hypodermis (**Figure 1A**).

A specialized cell wall feature of roots is the Casparian strip, a hydrophobically impregnated cell wall band between adjacent endodermal cells that seals the inside of the root from the outside (Geldner, 2013a). This barrier blocks the diffusion of harmful xenobiotics (exogenous toxic substances) from the soil into the vasculature and the leakage of nutrients in the reverse direction. Hence, while a large part of the nutrient and water transport pathway between the epidermis and the vasculature can proceed through the apoplast (along and across the cell walls), at the level of the endodermis, the nutrients have to pass the plasma membrane by active selective uptake, representing an effective filtering mechanism which is exemplified by the polar localization of nutrient influx and efflux carriers at the outer and inner side of endodermal cells, respectively (Ma et al., 2007; Alassimone et al., 2010; Takano et al., 2010).

While the first description of the Casparian strip dates back to 1865 (Caspary, 1865), its development has only recently been addressed at the molecular level in the non-mycorrhizal model species *Arabidopsis thaliana* (Geldner, 2013b). Thanks to mutants affected in the formation of the Casparian strip (Roppolo et al., 2011; Lee et al., 2013), the physiological significance of this specialized cell wall domain can now be addressed functionally in *Arabidopsis*. Similar mutants in AM-competent plant species will allow to address the question whether the Casparian strip could potentially contribute to the restriction of AM fungal colonization to the cortex.

Although plant cells are surrounded by cell walls, they are by no means isolated from each other. Thin cytoplasmic connections, referred to as plasmodesmata, bridge the walls between neighboring cells and allow for the exchange of signals and nutrients (Lucas et al., 2009). The plasmodesmata play an important role in root development by transporting the transcription factor SHORT ROOT from the vascular stele to the endodermal cell layer (Petricka et al., 2012). Furthermore, the plasmodesmata contribute to symplastic nutrient transport between the epidermis and the vasculature.

## SECONDARY DEVELOPMENT OF THE ROOT

The fine root hairs are restricted to a stretch of a few millimeters to centimeters behind the root tip. At later stages of development, and as a result of progressive damage of the delicate epidermal cells, the entire epidermis (including the root hairs) degenerates and the hypodermis becomes the new skin of the root (Meyer and Peterson, 2013). In these older parts of the root, AM can functionally replace the root hairs by the long hyphal extensions of the fungus (Smith and Read, 2008). In cases of perennial plants, the root shows secondary lateral growth as in the aerial stem (Chaffey, 2013). Interestingly, also herbaceous plants like *Arabidopsis thaliana* exhibit secondary root growth, which involves the generation of a cambial layer in the vascular stele that will form xylem to the inside and phloem to the outside, while the outer cell layers (endodermis, cortex, hypodermis, and epidermis) are shed (Dolan et al., 1993).

Besides the developmental aspects of secondary root development, the root is highly plastic to adapt to various environmental conditions such as drought anoxia, salinity etc. A distinct developmental program is triggered by anoxia (caused by flooding), when the roots form longitudinal air channels (aerenchyma) along the root which allow the transport of oxygen from the aerial parts of the plant to the root tips (Yamauchi et al., 2013). Formation of aerenchyma is associated with programmed cell death of cortical cell files, which causes these cell tiers to collapse and give rise to distinct tubular cavities along the root (Yamauchi et al., 2013). The root system also responds to nutrient availability in the soil. If nutrients are distributed heterogeneously, the roots explore the rich patches with intensely branching lateral roots. A genetically fixed adaptation of this kind is the formation of cluster roots, which are designed to the local exploitation of nutrients in the soil (Neumann and Martinoia, 2002). At a finer scale, the formation of lateral roots is stimulated by microbial signals (Olah et al., 2005; Maillet et al., 2011), whereas the development of entire mycorrhizal root systems is rather decreased relative to the shoot, conceivably as a consequence of the highly efficient role of the AM fungus in nutrient acquisition (Nouri et al., 2014).

## A ROLE FOR CELL WALLS IN SYMBIONT RECOGNITION AND HOST SPECIFICITY?

Since the cell walls are the outermost boundary of the root, they are the first interface for interactions with soil-borne microbes. Based on this notion, it has early been postulated that interactions of the cell walls between plants and microbes may play an important role in symbiotic interactions. Indeed, genetic analysis in legumes and rhizobia have led to the discovery that interactions between components of the host apoplast and extracellular determinants of the rhizobial partner can influence host range and symbiotic compatibility.

Rhizobia produce surface-bound and secreted polysaccharides such as lipopolysaccharides (LPS; consisting of carbohydrate backbones with lipid side chains; Becker et al., 2005), exopolysaccharides (EPS), and capsid polysaccharides (KPS) that influence the development of RNS. Some bacterial mutants in LPS are unable to infect their host plants, an effect that can be complemented either with co-inoculation with bacteria that provide the missing LPS, or with purified LPS (Mathis et al., 2005). Besides the function of LPS at early stages of the interaction, later phenotypes during infection indicate that LPS plays a role in suppression of defense and promotion of compatibility (Margaret et al., 2013). Since bacterial polysaccharides can affect the host range of rhizobia, they have been proposed to contribute to host specificity (Wang et al., 2012a). Similar functional evidence was obtained with mutants in EPS (Niehaus and Becker, 1998).

Bacterial polysaccharides can be bound by plant lectins with a certain degree of specificity (De Hoff et al., 2009). Lectins of legumes are localized to the tips of root hairs where the rhizobia first attach and are subsequently incorporated (Diaz et al., 1995; van Rhijn et al., 1998). Lectins were therefore proposed to play a role as determinants of host specificity (Bohlool and Schmidt, 1974). Indeed, it was shown that transformation with heterologous lectins extended the ability of the transgenic host to interact with the cognate symbiont of the gene donor, thereby increasing the host range (Diaz et al., 1989; van Rhijn et al., 1998). Although lectins can influence the host range they are currently thought to promote the progression of infection and to sustain compatibility, rather than being involved in early communication (De Hoff et al., 2009). With their ability to bind bacterial cells to the root hairs, they perhaps promote the communication with diffusible molecules and therefore could enhance signaling with flavonoid/nod factor (see below).

In the case of AM, the epidermal cell walls of the host can stimulate hyphopodium formation (see The Infection Process: Overcoming the Cell Wall), however, it is at present not clear to which degree this effect is relevant for host range determination and compatibility (see below). The fact that the development of AM is associated with the induction of lectin genes indicates that compatibility of the partners may also involve lectins in this fungal symbiosis (van Rhijn et al., 1998; Frenzel et al., 2005; Valot et al., 2005). Taken together, these examples show that cell wall interactions can modulate compatibility and therefore affect host specificity, although symbiotic communication relies primarily on diffusible signals (see below).

## DIFFUSIBLE SIGNALS IN ROOT SYMBIOSES

Due to their immobility and short range of action, cell wall bound agents such as LPS can only play a limited role in the soil, in particular at the early stages of the interaction before the partners have established a first physical contact with each other. Indeed, early experiments had shown that the growth and morphogenesis of AM fungi is influenced by diffusible factors emanating from host roots (Gianinazzi-Pearson et al., 1989; Giovannetti et al., 1994, 1996). Partial purification (Buée et al., 2000) and chemical identification (Akiyama et al., 2005) revealed that the stimulatory agent is represented by strigolactone (SL) that is exuded from host roots. SLs comprise a family of chemically related compounds (Xie et al., 2010) which have originally been identified as stimulants of seed germination in parasitic weeds such as *Striga*, and which later turned out to function as phytohormones in shoot development (Gomez-Roldan et al., 2008; Umehara et al., 2008).

Strigolactones may be the factor that causes chemotropic growth of AM fungi toward host roots (Sbrana and Giovannetti, 2005). In *Petunia*, SL is exuded by the ABC transporter pleiotropic drug resistance 1 (PDR1) from hypodermal passage cells (Kretzschmar et al., 2012), indicating that chemotropism could potentially occur at a short range on the root surface. PDR1 expression is enhanced by phosphate starvation but remains confined to hypodermal passage cells. SLs stimulate fungal metabolism resulting in increased hyphal branching (Besserer et al., 2006), conceivably increasing the chances of interaction with the host. However, the fact that SL-defective plants are still colonized to a considerable degree (Gomez-Roldan et al., 2008; Breuillin et al., 2010), and that fungal development is not affected in an obvious way Kretzschmar et al. (2012) suggests that SLs are not an absolute requirement for AM symbiosis. Conceivably, in the absence of SLs, other root-borne factors such as flavonoids may stimulate AM fungal growth and infection (Abdel-Lateif et al., 2012).

In the case of RNS, the chemical communication between the plant host and the bacterial symbiont has been particularly well studied thanks to the excellent genetic tools in both symbiotic partners (Oldroyd et al., 2011). Legume roots release flavonoids, that attract rhizobia chemotactically. The flavonoids are recognized in rhizobia by the NodD receptor proteins, which act as transcription factors that induce a suite of bacterial *nod* genes. These encode enzymes which produce specific symbiotic signals, the nod factors (NFs), chitin-related oligomers of *N*-acetylglucosamin of a length of 4–5 units with a fatty acid chain and various additional substitutions (Oldroyd, 2013). These lipochitooligosaccharides (LCO) trigger in the plant host the activation of a dedicated symbiosis signaling pathway, which is referred to as common symbiosis signaling pathway (further referred to as common SYM pathway), because it is involved in both, nodulation and AM (Oldroyd, 2013). Activating the common SYM pathway induces a rhythmic calcium signal, referred to as calcium spiking, which in turn triggers symbiosis-related gene expression and a cascade of morphogenetic events which result in the development of a new organ, the nodule, that hosts the symbiotic bacteria. The specificity in the interaction between the diffusible signals, flavonoids and NFs, and their respective receptors, NodD proteins and NF receptors (NFR), are thought to represent the basis of host specificity in RNS (Wang et al., 2012a).

Recently, AM fungal signals where discovered that are remarkably similar to NFs and are therefore further referred to as myc factors. They consist of LCO (Maillet et al., 2011), or even of bare short-chain chitin oligomers that can trigger signaling through the common SYM pathway (Genre et al., 2013). The latter finding could potentially resolve a long-standing conundrum of AM, their very low host specificity, since chitin-derived signals are unspecific. However, this mode of action would require additional signals from AM fungi that allows the host to distinguish them from other fungi with chitinous cell walls.

Two recent reports on the AM mutants *reduced arbuscular mycorrhiza1* (*ram1*), and *ram2* in *Medicago truncatula* shed new light on an interesting aspect of AM development. *RAM1* encodes a GRAS-type transcription factor that is highly induced in mycorrhizal roots (Gobbato et al., 2012). One of the functions of *RAM1* is to activate *RAM2*, which in turn encodes a glycerol-3-phosphate acyl transferase (GPAT) that is also highly induced during AM symbiosis (Wang et al., 2012b). The similarity of the phenotypes of *ram1* and *ram2* indicates that the main function of *RAM1* is to induce *RAM2*. GPATs catalyze the addition of a fatty acid moiety onto glycerol-3-phosphate, thereby generating an intermediate for lipid biosynthesis (both for storage and as membrane constituents) and for cutin/suberin biosynthesis. Whereas lipid biosynthesis is quite well characterized, the production of the extracellular cutin or suberin layer is still only partially understood (Pollard et al., 2008; Yeats and Rose, 2013). The following questions remain to be answered: Which are the intermediates secreted from the cytoplasm and how do they pass the plasma membrane? How are the intermediates linked to a 3-dimensional network in the apoplast and what is the exact structure of this network? Based on the similarity to GPATs of *Arabidopsis thaliana* it has been hypothesized that RAM2 contributes to the production of unknown cutin precursors (C16 and C18 hydroxy fatty acids) that are essential for AM (Wang et al., 2012b). Since the number of hyphopodia is reduced in *ram2* mutants, one of the potential interpretations has been that *ram2* mutants are defective in the production of a signaling compound that stimulates hyphopodium formation (Wang et al., 2012b). Surprisingly, appressorium formation by the oomycete pathogen *Phytophthora palmivora* was affected as well, suggesting that despite their very far phylogenetic distance the mycorrhizal fungus and the oomycete are stimulated by similar mechanisms (Wang et al., 2012b).

Interestingly, GPAT6 from *Arabidopsis thaliana* was able to complement the *Medicago ram2* mutant. GPAT6 is involved in cutin biosynthesis in *Arabidopsis* petals, suggesting that *ram2* function in *M. truncatula* could also be related to cutin biosynthesis. Indeed, the increase of GPAT activity by overexpression of *RAM2* in *Arabidopsis* leaves caused an increase in the cutin content of some C16 and C18 components in the leaf cutin layer. However, the fact that roots do not have a cutin layer suggests that an intermediate of cutin biosynthesis could regulate the interaction between AM fungi and plants. Indeed, roots contain several hydroxy fatty acids that normally constitute the cutin layer (Wang et al., 2012b), and the levels of some of these are reduced in *ram2*. A resulting conundrum is the fact that the mutation in the *RAM2* gene causes the substrate of the reaction (fatty acids) to decrease rather than the product (acyl glycerol). Along the same lines, the *ram2* mutant was complemented chemically with the substrate of the GPAT reaction rather than with its product (Wang et al., 2012b). In this context, it is interesting to note that hydroxy fatty acids can stimulate hyphal branching of AM fungi (Nagahashi and Douds, 2011). Taken together, some components of the cutin biosynthetic pathway are involved in the establishment of AM, however, further research is required to establish the exact nature of the molecules involved, and their function in symbiosis.

## THE INFECTION PROCESS: OVERCOMING THE CELL WALL

After legumes have established the first physical contact with the rhizobial partner, the root hairs undergo a characteristic morphogenetic change referred to as root hair curling, which results in the formation of a coil in which a bacterial micro-colony is trapped. This phenomenon represents a specific growth response of the root hair cell, brought about by reorientation of tip growth (Gage and Margolin, 2000). The orientation of root hair tip growth is thought to be controlled primarily by the direction of vesicle trafficking at the tip, which in turn is regulated by the actin cytoskeleton (Ketelaar, 2013). An active role of the plant cell in this process is evident from the fact that progression of the infection thread precedes bacterial migration (Fournier et al., 2008). Similarly, a signal from the epidermis triggers organogenesis in the cortex before the bacterial endosymbiont has reached these region of the root (Rival et al., 2012).

In the case of AM development, the fungus forms infection structures, from which penetration of the epidermis is initiated. These infection structures were initially termed appressoria in analogy to the infection structures of leaf pathogens that force their way through the cuticle and the outer cell wall by a combination of lytic enzymes and physical force (Deising et al., 2000). However, in the absence of evidence for the generation of physical force, they have recently been renamed to hyphopodia (hyphal foot). Indeed, based on the active role of the plant in infection, they function as “a foot in the door” rather than as a strong tool to force the door open. Experiments with isolated cell walls showed that the outer epidermal cell walls of the host *Daucus carota* (carrot) elicited hyphopodium formation in AM fungi, whereas cell walls of its vasculature, or epidermal cell walls of the non-host *Beta vulgaris* (sugar beet) did not (Nagahashi and Douds, 1997). Similar results were obtained by analysis of AM fungal growth patterns on intact roots of the non-host lupin (Giovannetti et al., 1993). Hence, the formation of hyphopodia requires stimulation by the host, which in this case involves specific physico-chemical stimulation. However, a purely physical (thigmotropic) stimulus as in the case of the appressoria of some pathogens (Deising et al., 2000) was excluded based on the fact that AM fungi do not form hyphopodia on artificial surfaces (Giovannetti et al., 1993).

The fact that AM fungi can form hyphopodia on isolated cell walls of host plants suggests that the fungus can recognize specific cell wall features (Nagahashi and Douds, 1997). Consistent with a central role for cell wall features in hyphopodium formation, SL-deficient plants can be colonized by AM fungi and appear morphologically normal, although the degree of colonization is significantly reduced (Gomez-Roldan et al., 2008; Breuillin et al., 2010; Kretzschmar et al., 2012). Hence, SLs are not an indispensable prerequisite for AM infection. Similarly, mutants in the common SYM pathway induce hyphopodium formation in the fungus, sometimes even at higher rates than in the wild type, although the hyphopodia often exhibit an aberrant morphology (Novero et al., 2002; Demchenko et al., 2004). In rice, which forms three root types (see Structure of Cell Walls in Roots), hyphopodium formation is restricted to the CR and large lateral roots, indicating that the SLRs lack a stimulating signal. Taken together, these observations indicate that features of the cell wall play an important role in hyphopodium formation.

**FIGURE 2:**
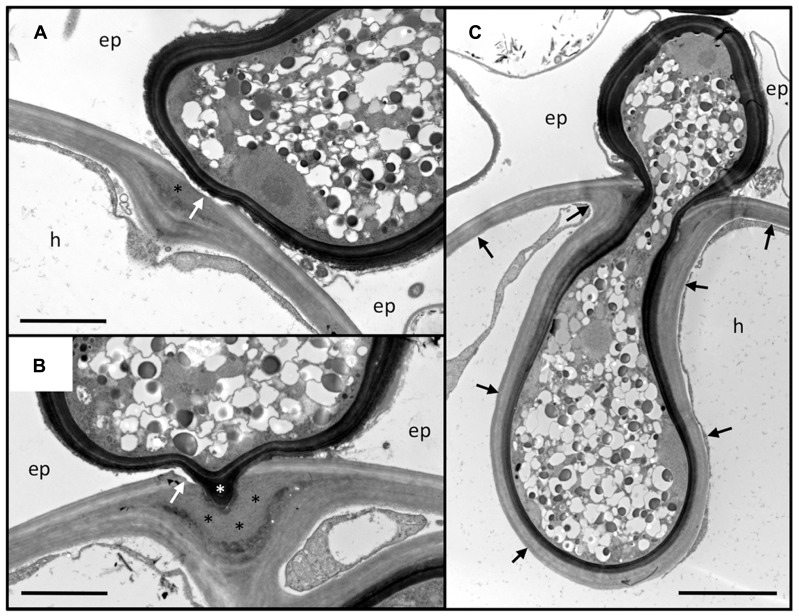
**Penetration of the outer root cell layers of *Petunia hybrida* by *Rhizophagus irregularis*.** Serial sections of an infection site at the level of the hypodermis. **(A)** Section through the hyphopodium (surrounded by a dark cell wall), that has entered between two adjacent epidermal cells (ep), and attached to a hypodermal cell (h). Note the alterations of the cell wall at the penetration site. The primary wall (light staining) is perforated (white arrow), whereas the secondary wall invaginates into the cellular lumen. In between, an interstitial matrix has been formed (asterisk). **(B)** Section close to the penetration hypha. The fungus has penetrated the primary wall (white arrow), whereas the secondary (inner) layers of the wall yield to the fungal penetration hypha (white asterisk). A thick layer of interstitial matrix material (black asterisks) has accumulated between the penetrated outer wall and the invaginated inner wall. **(C)** Median section through the penetration hypha. Note the inward deformation of the primary cell wall. The secondary wall has invaginated and is continuous with the original secondary wall (black arrows). ep, epidermal cell; h, hypodermal cell. Size bar 2 μm in **(A,B)** and 5 μm in **(C)**.

Hyphopodia are often formed in the groove between neighboring epidermal cells (**Figures 1A** and **2C**), from where the fungus either invades the neighboring epidermal cells or proceeds to penetrate the hypodermis. Evidence from *in vivo* imaging with fluorescently labeled cellular markers has shown that root cells actively prepare for infection and dictate the spatio-temporal progression of AM fungi infection through a structure referred to as prepenetration apparatus (PPA; Genre et al., 2005, 2008). The PPA is formed exactly below the hyphopodium, indicating that the plant cell can precisely locate the position of the hyphopodium. In plants that have a dimorphic hypodermis (see Anatomy of Plant Roots) penetration proceeds preferentially through hypodermal passage cells (Sharda and Koide, 2008). Conceivably, this is because of their greater permeability and weaker cell wall impregnation relative to the remaining hypodermal cells, and due to their release of SL (Kretzschmar et al., 2012). The mechanisms by which the cell walls are prepared to allow microbial penetration remain largely elusive, but are likely to involve cell wall softening agents (see below).

In apparent contrast to the evidence discussed above, a recent study reported the colonization of the non-host plant *Arabidopsis thaliana* by the AM fungus *Rhizophagus irregularis* (Veiga et al., 2013). This is surprising, in view of the fact that *Arabidopsis* misses many of the genes required for symbiotic signaling, and has not been reported to be colonized by AM fungi before. However, several characteristics of the interaction suggest that it does not represent a *bona fide* functional mycorrhizal interaction (Veiga et al., 2013). In general, colonized cells of *Arabidopsis* appeared to be dead or degenerating based on electron microscopic analysis, and the growth of colonized *Arabidopsis* plants was strongly inhibited (Veiga et al., 2013). The fact that the non-host *Arabidopsis* was invaded at all is probably due to the inoculum type, namely fungal hyphae emanating from mycorrhizal clover, which represents a very strong inoculum potential. Taken together, these aspects suggest that under the conditions of this study, the AM fungus acts like an opportunistic pathogen of *Arabidopsis*, rather than like a mutualistic symbiont.

## THE INTERACTION BETWEEN *Petunia hybrida* AND *Rhizophagus irregularis* – AN ANATOMICAL CASE STUDY

**FIGURE 3:**
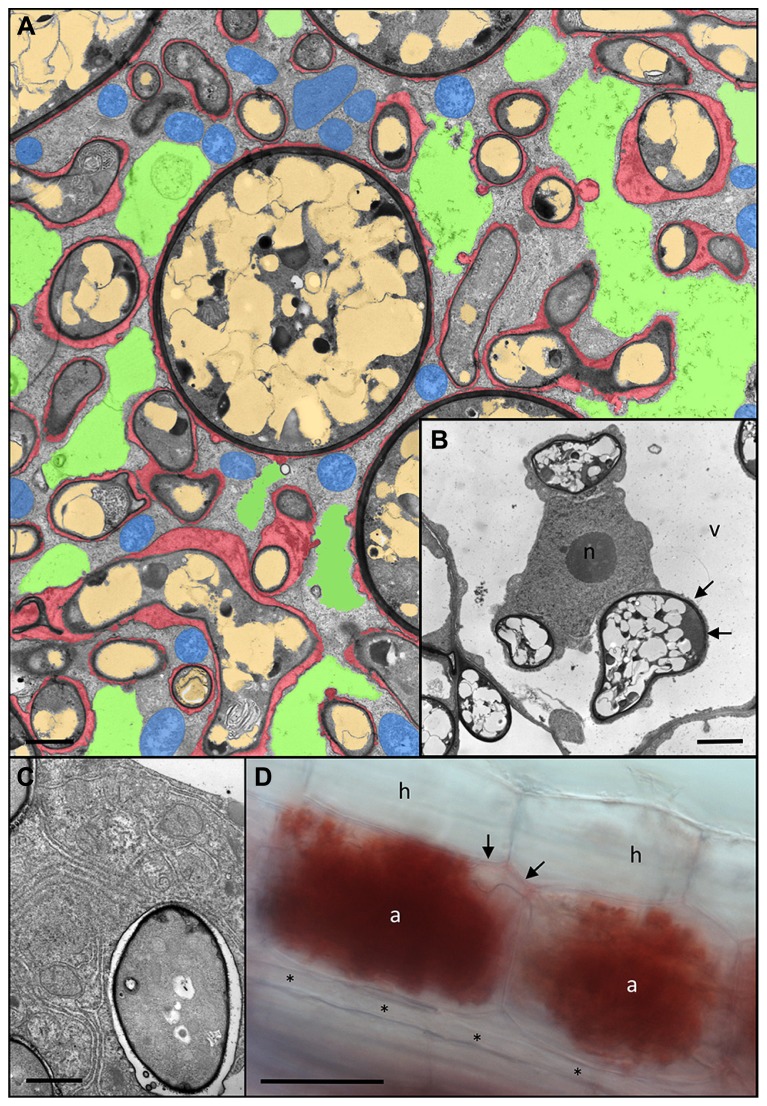
**Anatomy of *Rhizophagus irregularis* arbuscules and acidic nature of the periarbuscular space. (A)** Cortex cell of *Petunia hybrida* colonized by a fully developed arbuscule with hyphal branches of variable diameters. Remnants of the fragmented central vacuole are colored green, plant mitochondria are blue, fungal vacuoles are light brown, and the periarbuscular space is red (modified from Bapaume and Reinhardt, 2012). **(B)** Host nucleus (n) of a colonized cell between three fungal hyphae. Note the close association of the fungus with the host cytoplasm resulting in no distinguishable periarbuscular space between the fungal wall (dark layer), and the thin cytoplasmic layer (arrows) between the fungus and the host vacuole (v). **(C)** Close up of an individual fungal fine branch of an arbuscule. The fine branch is embedded in organelle-rich host cytoplasm with endoplasmatic reticulum and plastids. **(D)** A pair of mature arbuscules (a) sharing a joined trunk hypha (arrows). Staining with Neutral Red reveals the acidic nature of the periarbuscular space, whereas the trunk hypha shows only weak staining, and a longitudinal intercellular hypha (asterisks) is completely devoid of staining. a, arbuscule; h, hypodermal cell; n, host nucleus; v, host vacuole. Size bar 2 μm in **(A,B)**, 1 μm in **(C)**, and 50 μm in **(D)**.

*Petunia hybrida* is a powerful experimental model system for forward and reverse genetic analysis (Gerats and Vandenbussche, 2005), and has been used successfully for the analysis of genetic and nutritional regulation of AM (Breuillin et al., 2010; Feddermann et al., 2010; Nouri et al., 2014). Here, we describe the infection process of the AM fungus *Rhizophagus irregularis* (formerly *Glomus intraradices*) in *Petunia hybrida* with the focus on the events at the level of the cell wall during penetration of the hypodermis and in the established symbiosis (**Figures 2** and **3**). Since transmission electron microscopic (TEM) technology is destructive and can therefore not represent serial time course stages as in confocal microscopy (Genre et al., 2005, 2008), we provide a spatial gradient of cell wall modification at three different distances from a successful fungal entry point (**Figure 2**).

Usually, fungal hyphopodia insert between the adjacent anticlinal walls of two neighboring epidermal cells (**Figure 2**). Subsequently, the fungus invades the subtending hypodermal cell. In *Petunia*, the hypodermal cell layer is particularly thick-walled, with two clearly distinguished layers, an outer (primary) layer that is weakly stained by osmium tetroxide, and a thicker inner (secondary) layer that appears more electron-dense (**Figures 2A,B**). Penetration of this thick multilayer cell wall involves two mechanisms: firstly, the primary cell wall is dissolved locally, and secondly, the secondary cell wall invaginates to give way to the fungal penetration hypha. During hyphal growth, the secondary wall retains its original thickness around the entire hyphal coil indicating that the cell wall is continuously remodeled and extended (**Figure 2C**). The thick mass of interstitial matrix material (**Figures 2A,B**; black asterisks) may be involved in the softening of the cell wall, or may represent remnants of the dissolved cell wall. Hence, the outer part of the wall is degraded whereas the inner part is softened, remodeled, and extended, indicating that large amounts of new cell wall material are produced. Based on the fact that *Rhizophagus irregularis* does not have any cell wall lytic enzymes (Tisserant et al., 2013), and that penetration depends on recognition by the host (Parniske, 2008), it can be assumed that the cell wall alterations associated with fungal entry are orchestrated by the plant host.

Establishment of the intracellular symbiotic interface for nutrient exchange involves the colonization of the cellular lumen by arbuscules. These finely branched hyphal structures can occupy almost the entire cellular lumen, resulting in invasion and fragmentation of the central vacuole (**Figures 3A,B**). The fungal branches are surrounded by a thin layer of matrix material (red in **Figure 3A**). The finest hyphal branches are surrounded by a very thin layer of interfacial material and are embedded in cytoplasmic pockets rich in ER and organelles, indicative of strong metabolic and biosynthetic activity (**Figure 3C**).

## SYMBIOTIC INTERFACES

Nutrient exchange between the symbionts is regulated at the level of the plasma membrane of the microbe on the one side, and at the level of the host membrane that surrounds it on the other side, the peri-arbuscular membrane in AM, and the symbiosome membrane in RNS (Bapaume and Reinhardt, 2012). In between lies the periarbuscular space (in the case of AM), and the peribacteroid space (in the case of RNS), across which the nutrients are transferred, respectively. Collectively, this represents the symbiotic interface that controls communication and the exchange of nutrients between the cytoplasms of the two symbiotic partners.

In the case of RNS, the host membrane and all its contents (bacteroid and interface) are referred to as the symbiosome, hence representing functional units like the organelles with prokaryotic origin, the chloroplasts and the mitochondria (Kereszt et al., 2011). Based on the invaginated infection thread, which is related to the apoplast, the interface of the symbiosome could be compared topologically with the plant cell wall compartment, although, the symbiosomes represent rather specialized minivacuoles that harbor bacteroids and control their activities.

The feeding structures of AM fungi, the arbuscules, can be regarded as inverse haustoria that releases nutrients to the plant host. Haustoria are highly specialized feeding structures of parasitic fungi and oomycetes that serve to take up nutrients from the host (Deising et al., 2000; Panstruga, 2003; Yi and Valent, 2013). Active arbuscules are surrounded by an acidic compartment, as revealed by the accumulation of Neutral Red in the periarbuscular space (**Figure 3D**). Notably, the arbuscular trunk shows only light NR staining, whereas extracellular hypha are completely devoid of it. This indicates that the periarbuscular space may be sealed at the level of the trunk (Pumplin and Harrison, 2009), in order to avoid the loss of nutrients, and to maintain the acidic pH required to energize nutrient uptake from the interface (Gianinazzi-Pearson et al., 2000; Guttenberger, 2000). Similarly, the peribacteroid space represents an acidic compartment from which the plant host and the bacteroid can take up nutrients using the proton gradient as motive force (Pierre et al., 2013).

The periarbuscular space consists of loosely assembled cell wall polymers including cellulose, hemicellulose, pectin, and arabinogalactan/hydroxyprolin-rich proteins (Gianinazzi-Pearson, 1996; Balestrini and Bonfante, 2005). In addition, the symbiotic interface may contain some of the predicted extracellular proteins that are induced in AM and often restricted to cells with arbuscules (e.g., van Buuren et al., 1999; Journet et al., 2001). Notably, cells with arbuscules express specific cell wall remodeling proteins that may help establish the interface during the complex morphogenesis of the two symbiotic partners (Balestrini et al., 2005; Dermatsev et al., 2010; see below).

In functional nodules, the rhizobia undergo fundamental reprogramming, which often results in terminal differentiation of the prokaryote, i.e., it loses vital functions and becomes entirely dedicated to its symbiotic function, namely N-fixation. At this stage, they are referred to as bacteroids, and they are confined by a host-derived membrane that controls nutrient exchange and the fate of the endosymbiont. While the nature of the arbuscular interface has been studied primarily by immunocytochemistry and gene expression analysis (Balestrini and Bonfante, 2005), the composition of the interface in symbiosomes has been addressed by proteomics, based on the fact that symbiosomes can be easily enriched and purified (Saalbach et al., 2002). However, a problem of this technique is that the interface and the symbiosome membrane tend to be contaminated from cytoplasmic bacteroid proteins. Nevertheless, this approach confirmed that the peribacteroid space in pea nodules contains a lectin that has earlier been recognized as a resident protein of the peribacteroid space (Kardailsky et al., 1996). An interesting case of secreted peptides that accumulate in the peribacteroid space are the nodule-specific cysteine-rich (NCR) peptides. NCR peptides cause terminal differentiation in bacteroids, indicating that the host uses them as a tools to manipulate the development of the endosymbiont (Van de Velde et al., 2010), in order to prevent it from overproliferation (Kereszt et al., 2011).

## TRAFFICKING AT THE SYMBIOTIC INTERFACE

The primary function of the symbiotic interface is nutrient exchange. The specialized function of the interface in AM is exemplified by the expression of transporters for phosphate (Rausch et al., 2001; Harrison et al., 2002; Yang et al., 2012), and ammonium (Guether et al., 2009b; Kobae et al., 2010; Koegel et al., 2013) in colonized cells. A pair of ABC transporters in the periarbuscular membrane, STUNTED ARBUSCULE (STR), and STR2, that are functionally conserved between the legume *M. truncatula* and the cereal rice (Zhang et al., 2010; Gutjahr et al., 2012), may be involved either in the transport of a metabolically relevant substrate, or in signaling between the partners, however, until the identification of its substrate the function of STR and STR2 remains elusive. Furthermore, periarbuscular membranes carry ATPases for acidification of the symbiotic interface (Marx et al., 1982; Gianinazzi-Pearson et al., 2000).

Among the AM-related genes with high induction ratios in various plant species are proteases that are predicted to carry a secretion signal peptide (Takeda et al., 2007). These proteins are therefore expected to enter the protein trafficking pathway and to be delivered to the vacuole, the apoplast, or, potentially, to the symbiotic interface. The signal peptide of an AM-associated protease of the subtilase type has indeed been shown to confer localization to the periarbuscular space (Takeda et al., 2009). Silencing of this protease hampers AM development, indicating that it is involved in fungal growth or in symbiotic signaling (Takeda et al., 2009). However, its precise function in AM remains elusive.

Many genes induced during nodulation (nodulins) encode secreted proteins, for example the early nodulins (ENOD proteins) among which there are many prolin-rich extracellular proteins (Brewin, 2004). A detailed study of ENOD8 localization showed that it has multiple independent determinants of symbiosome localization, including the N-terminal signal peptide (Meckfessel et al., 2012). Interestingly, in non-symbiotic cells, ENOD8 is delivered to the vacuole. This suggests that either the symbiosome has vacuolar features, or that protein trafficking is rerouted to the symbiosome in symbiotic cells. Interestingly, a general rerouting of protein trafficking has been documented in AM symbiosis, where all membrane proteins expressed from the symbiosis-specific PT4 promoter were localized to the periarbuscular membrane (Pumplin et al., 2012). The surprising conclusion of these experiments is that protein trafficking in mycorrhizal cells is not based on the peptide sequence but on the timing of gene expression. Taken together, these results indicate that protein trafficking toward the symbiotic interface may be proceed by default in symbiotic cells. Components with a potential role in secretion to the symbiotic interface include the symbiosis-induced SNARE proteins VAMP72d and VAMP72e which are localized to the host membrane at the symbiotic interface (Ivanov et al., 2012).

## CELL WALL REMODELING IN SYMBIOSIS

Invasion of host cells by microbes requires local softening of the cell wall at the point of penetration. Cell wall softening can be achieved either by partial degradation of the wall polymers, or by weakening of the intermolecular interactions between the cell wall polymers (Cosgrove, 2000a). Evidence from TEM analysis of the interaction between *Petunia hybrida* and *Rhizophagus irregularis* suggests that both mechanisms may be involved simultaneously (**Figure 2**).

Conceptually, the cell wall lytic or loosening agents could be encoded by genes of the host or the microbe. *Rhizobium leguminosarum* possesses a cell-wall-bound β-1,4 endoglucanase (cellulase CelC2), required for formation of infection threads and for successful bacterial infection, suggesting that the bacterial endosymbiont uses the cellulase to breach the cell wall of root hair tips (Robledo et al., 2008). However, early work has shown that local cell wall loosening can also occur in the absence of the microbial partner during the formation of pre-infection threads, indicating that in this case the loosening agent is encoded by the host (van Brussel et al., 1992; van Spronsen et al., 1994). Indeed, *Lotus japonicus* has an inducible pectate lyase gene that is required for rhizobial infection (Xie et al., 2012).

In contrast to rhizobia, the AM fungal model species *Rhizophagus irregularis* appears to lack genes with a predicted cell wall degrading, modifying or remodeling activity (Tisserant et al., 2012, 2013). The same is true for the obligate biotrophic pathogen *Blumeria graminis* (Spanu et al., 2010). Along the same lines, the genomes of the ectomycorrhizal fungi *Laccaria bicolor* and *Amanita bisporigera* have greatly reduced numbers of potential cell wall degrading genes compared to saprophytic fungi (Martin and Selosse, 2008; Martin et al., 2008; Nagendran et al., 2009). The reduction or elimination of genes encoding cell wall-modifying proteins in biotrophic microbes may represent a selective advantage given the fact that plants have extremely sensitive detection mechanisms for such microbial activities and react with a defense response (see below). Hence, in the case of AM fungi, the wall loosening activity appears to be encoded primarily or entirely by the host.

In contrast to lytic enzymes, expansins act by loosening cell walls non-enzymatically, presumably by allowing cellulose microfibrils to creep relative to the surrounding wall matrix in a fashion analogous to a lubricant. (Cosgrove, 2000b). Expansin is induced during RNS (Giordano and Hirsch, 2004) as well as in AM (Balestrini et al., 2005; Dermatsev et al., 2010). Indeed, the silencing of tomato expansin interferes with AM, indicating that expansin activity may be required for proper arbuscule development (Dermatsev et al., 2010).

Apart from the expansins, several symbiosis-related transcripts encode host genes with a potential role in cell wall remodeling, for example xyloglucan endotransglycosidase induced in colonized cells of AM (van Buuren et al., 1999). In AM, the formation of the PPA is characterized by high vesicular activity (Genre et al., 2008) and by induction of cell wall modifying proteins such as cellulose synthase and expansin-like proteins (Siciliano et al., 2007). Conceivably, these proteins are targeted to the PPA and released to prepare the cell wall for penetration.

## DEFENSE RESPONSES IN SYMBIOSIS

Plants have very sensitive perception mechanisms to detect microbial infection and to react with a defense response to inhibit microbial growth (Jones and Dangl, 2006). The signal molecules derived from pathogens are referred to as pathogen-associated molecular patterns (PAMPs) and in cases where rather unspecific signals from a wide range of microbes (e.g., chitin) are concerned, the signals are referred to as microbe-associated molecular patterns (MAMPs; Boller and Felix, 2009). Often, the microbe is detected via its direct or indirect effects on the host cell wall. Many pathogens release hydrolytic enzymes that weaken the cell wall by degrading the polymers in the cuticle (cutinase) or the cell wall (xylanase, glucanase etc.), thereby releasing monomers that are perceived by plants at concentrations in the nanomolar range (Boller and Felix, 2009). In addition, plants can detect metabolites that emanate from damaged tissues and therefore serve as indicators of damage [damage-associated molecular patterns (DAMPs)]. Some MAMPs can be produced as a result of defense-related hydrolytic enzymes of the host (chitinase, glucanase) that release oligomers from the microbial cell wall (Boller and Felix, 2009).

It has long been suggested that mutualistic microbes also release or induce MAMPs and DAMPs, and indeed, symbiosis is often associated with the induction of defense markers (Blee and Anderson, 2000; Lambais, 2000; Shaul et al., 2000; Gianinazzi-Pearson and Smith, 2003; Lopez-Gomez et al., 2012; Zamioudis and Pieterse, 2012). Although it has often been observed that defense responses in AM are only transient (Gianinazzi-Pearson et al., 1996), some homolog of defense marker genes remain strongly induced during symbiosis raising the question whether they can be regarded as defense markers (see below). Cellular and biochemical defense responses, such as the production of reactive oxygen species or the hypersensitive response, could potentially interfere with symbiosis. A possible mechanisms to counteract such defense responses and to promote compatibility in symbiosis would be to remove the signals that trigger them. Such a function has been proposed for host-derived chitinase that could potentially degrade fungal chitin oligomers in mycorrhizal associations (Salzer and Boller, 2000), or even transform them into short chain chitin oligosaccharides that serve as symbiotic signals (Genre et al., 2013).

Pathogens have evolved efficient tools, collectively referred to as effectors, to suppress defense responses. Effectors are delivered to the host to inhibit defense at various levels from signal perception to the induction of the defense response (Stergiopoulos and de Wit, 2009; Deslandes and Rivas, 2012). Several recent studies have revealed similar mechanisms in mycorrhizal fungi and rhizobia (Kloppholz et al., 2011; Plett et al., 2011; Zhang et al., 2011; Xin et al., 2012; Okazaki et al., 2013). The large number of symbiosis-induced predicted secreted proteins in the genomes of the ectomycorrhizal fungus *Laccaria bicolor* (Martin and Selosse, 2008; Martin et al., 2008) and the AM fungus *Rhizophagus irregularis* (Tisserant et al., 2012, 2013) indicates that still many mycorrhizal effectors remain to be discovered. Interestingly, NF-defective rhizobia can establish symbiosis using effectors delivered by the type-three secretion system (Okazaki et al., 2013). This suggests that NFs and myc factors could be regarded as specialized effector-like tools to promote compatibility during nodulation and AM development, respectively, a concept that has received recent support from *Arabidopsis thaliana* (Liang et al., 2013). In this context, it is interesting to note that legume mutants with a defect in the common SYM pathway, which is required for NF/myc factor signaling, mount a distinct defense response upon inoculation with AM fungi (Gollotte et al., 1993, 1994). Hence, one function of the common SYM pathway is to suppress defense in symbiotic interactions.

## ROLE OF PR PROTEINS IN SYMBIOSIS

Plant defense is often accompanied by the induction of pathogenesis-related (PR) proteins that are thought to have antimicrobial activity for instance by hydrolyzing components of microbial cell walls (van Loon et al., 2006). Many PR proteins are secreted and reside in the apoplast (van Loon et al., 2006). Interestingly, homolog of PR proteins are also induced during AM (Liu et al., 2003; Manthey et al., 2004; Güimil et al., 2005; Hohnjec et al., 2005; Grunwald et al., 2009; Guether et al., 2009a; Breuillin et al., 2010). Transcriptomic analysis in *Petunia hybrida* revealed that numerous PR protein homolog were undetectable in non-mycorrhizal roots, whereas others showed strong induction ratios over their constitutive basal expression levels (Breuillin et al., 2010). Notably, they were induced not just transiently at the onset of the interaction, but in fully developed mycorrhizal roots, indicating that they are more than markers of a transient initial defense response.

Based on the observation that AM fungi appear to be generally insensitive to high constitutive PR gene expression in plants (Vierheilig et al., 1993; Gianinazzi-Pearson et al., 1996), it could be argued that the induction of PR genes contributes to a generally induced disease resistance status in mycorrhizal plants (Pozo and Azcon-Aguilar, 2007). Alternatively, AM-specific PR gene isoforms could play specific roles in symbiosis. Indeed, it has been shown for the example of Chitinase III in *M. truncatula* that individual isoforms are expressed specifically during defense and mutualism, respectively (Salzer et al., 2000). Although a role for AM-related chitinase III has been suggested based on its stimulatory effect on spore germination (Elfstrand et al., 2005), its role in established mycorrhizal roots remains to be explained.

**FIGURE 4:**
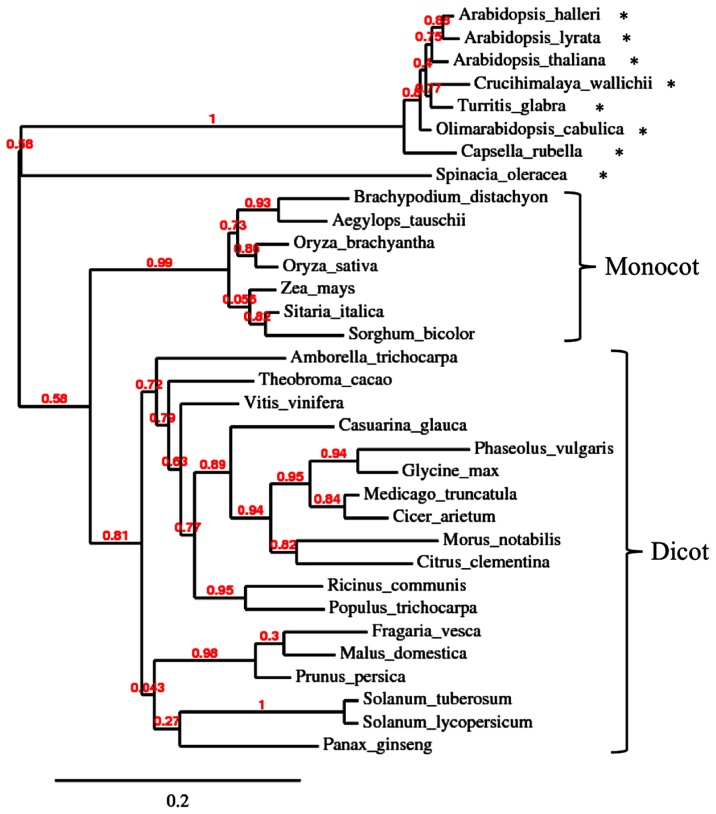
**Phylogenetic analysis of type III chitinase in higher plants.** Unrooted phylogenetic tree of chitinase type III of various angiosperm species. Protein sequences were retrieved by pblast from NCBI using *Medicago truncatula* chitinase III-3 (AY238969.1) as query. For each species, the first hit was taken for phylogenetic analysis. Non-mycorrhizal species are marked with an asterisk. Note that the non-mycorrhizal *Brassicacea* and spinach (*Spinacia oleracea*) form an outgroup relative to the more conserved chitinases of the mycorrhizal monocot and dicot species.

A symbiosis-specific role of symbiosis-related PR protein homolog is conceivable in cases, where they are encoded by genes that are conserved to a higher degree in AM-competent plant taxa than in non-symbiotic species. For example, the AM-specific chitinase III isoform of *M. truncatula* has close homolog in mycorrhizal dicots as well as monocots, whereas the AM-incompetent *Brassicaceae* and the *Chenopodiacae* species spinach have more distant homolog (**Figure 4**). The fact that this chitinase isoform is particularly well conserved among AM-competent taxa indicates that it is under selective pressure for a function in AM. For example, chitinase could act by generating chitin oligomers that are involved in AM signaling (Genre et al., 2013). In this context, it is interesting to note that nodulation also involves chitinases that are thought to influence NF signaling (Goormachtig et al., 1998; Tian et al., 2013).

## FOOD FOR THOUGHT: THE INVOLVEMENT OF THE CELL WALL COMPARTMENT IN NUTRITION OF SYMBIOTIC MICROBES

Arbuscular mycorrhiza fungi are obligate biotrophs, i.e., they rely on living root tissues for survival, implying that they depend on the supply of some plant metabolites that cannot be provided in the form of plant extracts. Whether this concerns the acquisition of reduced carbon or of some other vital factor is unknown. Also, it is not clear whether AM fungi acquire their nutrients over the arbuscules or along their intercellular hyphae. The fact that ectomycorrhizal fungi can live without intracellular feeding structures indicates that the apoplast of roots can provide sufficient extracellular resources to sustain a mycorrhizal network, although the formation of the dense Hartig net indicates that the efficient acquisition of these resources requires an extended hyphal surface.

Photoassimilates are transported through the phloem in the form of sucrose. In order to become available to fungal symbionts in the root cortex, the sucrose (or a derived carbohydrate) has to cross the endodermis symplastically and to be released to the apoplast or the symbiotic interface in the cortex. Based on circumstantial evidence, it has been suggested that AM fungi use hexoses as carbon source (Solaiman and Saito, 1997; Pfeffer et al., 1999; Douds et al., 2000). The fact that the genome of the AM fungal model species *Rhizophagus irregularis* does not contain any secreted invertases or sucrose transporters is compatible with the idea that this fungus relies entirely on the supply of hexoses from the plant (Tisserant et al., 2013), and the recent discovery of a versatile monosaccharide transporter (MST2) from *Rhizophagus irregularis* support this view (Helber et al., 2011). In the absence of efficient transformation protocols for AM fungi (Helber and Requena, 2008), the function of MST2 was addressed by expression of a silencing construct against the fungal gene in the host. The intimate interaction of the symbiotic partners over the symbiotic interface allows for efficient silencing of the fungal target gene, a method referred to as host-induced gene silencing (HIGS; Helber et al., 2011). The fact that MST2 is expressed both in arbuscules and in intercellular hypha suggests that hexose uptake could potentially proceed anywhere along the extended intraradical fungal network.

While the induction of an invertase in mycorrhizal cells of tomato is consistent with the model discussed above (Schaarschmidt et al., 2006), AM colonization could not be stimulated by increased invertase activity in transgenic tobacco, suggesting that under the conditions examined, hexose supply to the fungus is not limiting (Schaarschmidt et al., 2007). On the other hand, silencing of a cytoplasmic sucrose synthase (a sucrose cleaving enzyme) of *M. truncatula* interferred with both AM colonization and nodulation (Baier et al., 2007, 2010), pointing to a connection of sink strength and symbiosis. However, due to the pleiotropic phenotype of *SucS1* silencing, these results have to be interpreted with caution. Taken together, the nutrition of AM fungi remains far from clear, whereas it is commonly accepted that in RNS the prokaryotic endosymbiont is fed primarily by dicarboxylic acids (Yurgel and Kahn, 2004).

## CONCLUSION

As the outermost border of the plant body, the cell wall mediates many interactions with the environment of plants. Besides the multiple roles of the cell wall in basic plant life (growth, nutrient exchange etc.), the cell wall plays a central role in interactions with the biotic environment. While it represents the primary barrier against pathogens, it has to yield to permit the invasion of mutualistic microbes such as rhizobia and AM fungi. The cumulative evidence suggests that the plant itself plays a central role in the softening and remodeling of the cell wall during symbioses. Future work should address the molecular components involved in cell wall remodeling during endosymbiont infection, and further characterize the nature of the symbiotic interface during the different stages of the interaction.

## Conflict of Interest Statement

The authors declare that the research was conducted in the absence of any commercial or financial relationships that could be construed as a potential conflict of interest.
